# Methylglyoxal-Induced Retinal Angiogenesis in Zebrafish Embryo: A Potential Animal Model of Neovascular Retinopathy

**DOI:** 10.1155/2019/2746735

**Published:** 2019-04-17

**Authors:** Ying Li, Yantao Zhao, Shengmin Sang, TinChung Leung

**Affiliations:** ^1^Department of Geriatrics, Qi-Lu Hospital of Shandong University, Jinan, Shandong Province 250012, China; ^2^Julius L. Chambers Biomedical/Biotechnology Research Institute, North Carolina Central University, Kannapolis, NC 28081, USA; ^3^Laboratory for Functional Foods and Human Health, Center for Excellence in Post-Harvest Technologies, North Carolina A&T State University, Kannapolis, NC 28081, USA; ^4^Department of Biological & Biomedical Sciences, North Carolina Central University, Durham, NC 27707, USA

## Abstract

Methylglyoxal (MG) is an intermediate of glucose metabolism and the precursor of advanced glycation end products (AGEs) found in high levels in blood or tissue of diabetic patients. MG and AGEs are thought to play a major role in the pathogenesis of diabetic retinopathy. In order to determine if zebrafish is valuable to help us understand more about retinopathy, we evaluate if MG induces abnormal vascular change and angiogenesis in zebrafish in a short incubation period. We also used an inhibitor of VEGFR (PTK787) to explore the mechanistic role of VEGF in MG-induced pathogenesis. A transgenic *Tg(flk1:GFP)* zebrafish line was used, and the embryos were incubated with MG solution and in combination with glucose (to mimic hyperglycemia). Retinal vascular structure visible with fluorescence signal was imaged using fluorescence microscopy. The percentage of vascular area was calculated and found elevated in the MG treatment groups than that in the control group (*p* < 0.01) which indicated increased angiogenesis induced by MG treatment. PTK787 blocked the proangiogenic effects of MG treatment. This study suggests that MG has a potential proangiogenic effect via VEGF signaling in the retina of zebrafish embryos. Therefore, this zebrafish model may be used to study neovascular retinopathy.

## 1. Introduction

Diabetic retinopathy (DR) is the major microvascular complications of all forms of diabetes closely associated with long-term hyperglycemia [1]. It is a major cause of acquired blindness in people of working age all over the world [2–4]. The incidence rate of DR increases with the development of diabetes mellitus (DM). It develops in around 80% of patients of DM after they were diagnosed for 20 years [5]. The early stage of DR, nonproliferative diabetic retinopathy (NPDR), is characterized by a loss of pericytes of retinal capillaries, microaneurysm, and soft and hard exudates caused by increased retinal capillary permeability. All of the changes result in retina ischemia and nonperfusion area formation. Accompanied by increased expression of the vascular endothelial growth factor (VEGF) [6], proliferative diabetic retinopathy (PDR) occurs mainly characterized by neovascularization of the retina. PDR is the most serious complication and the leading cause of blindness in diabetic patients.

In addition, there are many other neovascular retinopathy, including age-related macular degeneration (AMD), retinopathy of prematurity (ROP), and retinal vein occlusion (RVO), leading to severe visual impairment with major pathological changes caused by angiogenesis. Therefore, the study of animal models of neovascular ophthalmopathy is of great significance.

Although many species of animal models have been used in the study of diabetes, such as mice, rats, rabbits, and dogs, there is no ideal animal model to mimic the clinical progression and pathology of DR in human, especially difficult to show the proliferative angiogenesis in limited time. Over the past decade, zebrafish has been proved to be a valuable vertebrate model to study the pathology of DR and visual diseases [7–11]. The significant advantages over the other traditional animal model include their small size, high fertility rate, and rapid development of the visual system [12, 13]. Zebrafish also shares many similarities with the retinal vasculature [14–17] and vascular pathology [18] in mammals. For studying angiogenesis-related disease, zebrafish has unique advantages over other animal models [18, 19].

Methylglyoxal (MG), a reactive alpha-dicarbonyl intermediate of glucose metabolism, is the precursor of advanced glycation end products (AGEs) found in high levels in blood or tissue of diabetic patients [20, 21]. AGEs can be derived from nonenzymatic glycation of proteins and are thought to play a major role in the pathogenesis of diabetic retinopathy [22]. MG induced endothelial cell dysfunction [23, 24] and has been proposed as a causative factor of retinal vascular injury [25, 26]. It is reported that MG-derived hydroimidazolone has been found in increased levels in the target organs and serum in animals and humans with type 2 diabetes [21, 27–29]. Moreover, patients with PDR had increased serum levels of MG-derived hydroimidazolone when compared to those with nonproliferative diabetic retinopathy [30]. Many evidence suggested that human ocular diseases such as glaucoma, cataract, diabetic retinopathy and age-related macular degeneration can be studied using the zebrafish [31, 32]. It suggested that MG may play an important role in the proliferative stages of DR. Jorgens and colleagues used zebrafish as a model to analyze early vascular effects and mechanisms of MG in vivo [33]. They found MG-induced angiogenesis from intersomitic blood vessels (ISVs) only within 4 days of treatment, independent of high glucose treatment. They demonstrated that incubation with MG rapidly increased the bioavailability of MG content in zebrafish embryos, confirming this experimental approach as a useful strategy in the zebrafish model to study potential effect of MG on retinopathy. Here, we try to address the burden of animal models which have extensive length of incubation period for clinical symptom to appear, especially the difficulty to show any proliferative angiogenesis in limited time, and we investigate if zebrafish embryo can be used to study abnormal vascular change in retina to resemble one of the aspects of the retinal defect in neovascular retinopathy. Since the hallmark of PDR is neovascularization that occurs at the vitreoretinal interface, the zebrafish embryos can be used to evaluate the diabetic vascular retinal defects of short-term exposure to methylglyoxal. However, the zebrafish embryo has a limitation that it is not an adult whereas PDR usually occurs in later stage of retinopathy in adults. In addition, the progression of PDR is a lengthy process; our zebrafish embryos are designed to evaluate acute responses of blood vessels to methylglyoxal, which may be beneficial for large-scale drug screen; however, the embryo model cannot completely recapitulate the vascular pathology induced by chronic hyperglycemia or methylglyoxal activation of the vessels in PDR. Nevertheless, we take advantage of the zebrafish model to evaluate the potential effect of MG on abnormal retinal angiogenesis in the embryos to establish a potential neovascular retinopathy model. During DR progression, increased local concentrations of VEGF are central to the etiology of proliferative DR [6]; therefore, we also used VEGFR inhibitor on MG-treated zebrafish to explore the mechanism of pathogenesis.

## 2. Methods

### 2.1. Fish Maintenance and Husbandry

A transgenic zebrafish line *Tg(flk1:GFP)* in AB background, expressing green fluorescent protein (GFP) in all vasculature including the retinal vasculature, was maintained on a 14/10-h (light/dark) photoperiod in Aquaneering and Aquatic Habitat zebrafish housing units. The zebrafish embryos were maintained at 28.5°C in 0.3X Danieau's solution (19.3 mM·NaCl, 0.23 mM·KCl, 0.13 mM·MgSO_4_, 0.2 mM·Ca(NO_3_)_2_, 1.7 mM·HEPES, and pH 7.0) [34]. All experimental protocols and procedures were approved by and conformed to the guidelines of the Animal Care and Use Committee of North Carolina Central University (Durham, NC) (NCCU IACUC Protocol # TCL-07-14-2008).

### 2.2. Drug Treatment

The zebrafish eggs were incubated with 500 *µ*M and 1000 *µ*M MG with or without 30 mM glucose starting from 10 hour-post-fertilization (hpf) to 4 day-post-fertilization (dpf). MG plus glucose treatment was to mimic the clinical condition of hyperglycemia in patients with diabetes. Likewise, GS4012 (1 *µ*M and 3 *µ*M) [35], a VEGF inducer, was used to produce retinal angiogenesis as the positive control. To study the effect of VEGF on MG-induced vascular retinopathy, we used 0.5 *µ*M PTK787 (Tocris Bioscience, Minneapolis, MN), an inhibitor of vascular endothelial growth factor receptor tyrosine kinase, from 1 to 4 dpf added into the medium containing MG and glucose. A dose dependence response study of various solutions (0.1–1 *µ*M) of PTK787 was conducted preliminarily to determine the proper concentration of PTK787 for angiogenesis observation. Because of the marked inhibition effect on MG-induced retinal vascular change with no other observable abnormality at 0.5 *µ*M PTK787, we used this concentration in subsequent analysis. During all the periods of experimentation, no obvious morphological toxicity was observed in the embryos when exposed to 1000 *µ*M MG, 30 mM glucose, 3 *µ*M GS4012, and 0.5 *µ*M PTK787. Survival rates for all groups were 100% at 4 dpf.

### 2.3. Observations and Fluorescence Imaging Analysis

**Figure 1:**
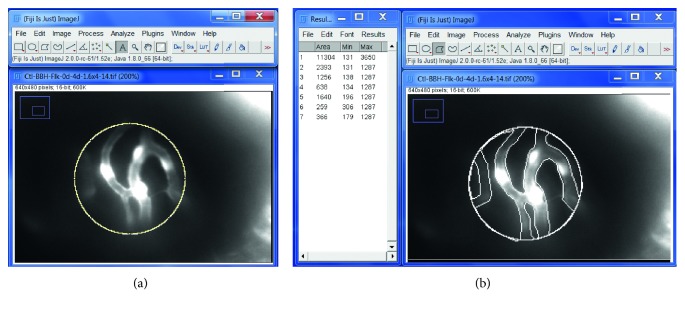
Quantification of the vascular area of retina at 4 dpf using Fiji-ImageJ software. An example of a retinal vascular image: use Fiji-ImageJ “area measurement” function under “Analyze” to measure the relative vascular vessel area over the retinal area. (a) Total retinal area by drawing a uniform circle of 120 pixel diameter = 11304 pixel area. (b) Draw and measure areas outside the vascular vessels = 2393 + 1256 + 638 + 1640 + 259 + 366 = 6552. Then, calculate % vascular vessel area of the retina = (11304 − 6552)/11304 × 100% = 42.0%. ^*∗*^Branch point of retinal vessels.

Before observations, 4 dpf zebrafish embryos were anaesthetized with 0.168 mg/mL tricaine (Sigma-Aldrich, Milwaukee, WI). Retina vascular fluorescence from 30 eyes/15 embryos per group was imaged and analyzed using MetaMorph TL for Olympus (Olympus, Center Valley, PA), MVX10 MacroView Fluorescence Microscope (Olympus, Center Valley, PA) and Hamamatsu C9300-221 high-speed digital CCD camera (Hamamatsu City, Japan), coupled with VAST BioImager Platform (Union Biometrica, Boston, MA), for automating the handling of zebrafish larvae prior to high-resolution imaging. The vascular area of retina at 4 dpf was quantitated using Fiji-ImageJ software (Figure 1). The exact position of eye fundus of zebrafish larvae was positioned in VAST Imager glass capillary under the fluorescence microscope, and the embryo was rotated until the eye position allows the imaging from central to peripheral regions of the retina; the position was standardized at the angle to allow the imaging around the eye fundus, basically equivalent in the midperiphery as described in Hamanaka's study [36]. Compared to the central large vessels near the optic disc of the posterior pole, the midperipheral area have higher density of blood vessel branches for better sensitivity to detect any proangiogenic effect. In terms of the field of view for the eye, the midperipheral region of the retina receives light from the 30° to 60° of the field of view, whereas the fovea/central region of the retina receives light from the center of the visual field, i.e., 5° visual angle. Our assay for detecting vascular changes in the retinal vessel is based on 2D fluorescent image at the midperipheral area.

### 2.4. Western Blot Assay of Proteins

Whole-protein lysates of zebrafish eyes were homogenized and extracted using RIPA buffer (25 mMTris-HCl, pH 7.6, 150 mM NaCl, 1% NP-40, 1% sodium deoxycholate, and 0.1% SDS; Thermo Fisher Scientific) supplemented with 1% protease inhibitor cocktail and 1% phenylmethylsulfonyl fluoride. 30 *μ*g protein samples were loaded onto a 10–12% sodium dodecyl sulfate polyacrylamide gel. Antibodies of VEGF (R&D System, Minneapolis, MN) and *β*-actin (Abcam, Cambridge, MA) and horseradish peroxidase-conjugated antibody (Cell signaling, Danvers, MA, USA) were used for chemiluminescent detection (Thermo Scientific, Rockford, IL, USA). The immunoblot bands were quantified by densitometry analysis, and the ratio to *β*-actin was calculated and presented, setting the values of control as one.

### 2.5. Statistical Analysis

Data of % vascular areas were expressed as average with standard error of the mean (±SEM). Statistical analysis was performed by one-way ANOVA, and then, the *p* values were calculated using Student's *t*-test.

## 3. Results

### 3.1. MG-Induced Vascular Changes in the Retina

It is known that early diabetic retinopathy is correlated with the pericyte loss of the retinal capillaries. Kim et al. suggested that MG induced apoptosis on bovine retinal pericytes and produced a progressive cytotoxic effect with increasing concentration (200 to 800 *µ*M) [37]. It was also demonstrated that external MG at the concentration of 200 or 500 *µ*M can rapidly increase MG level in tissues of live zebrafish embryos and MG can induce angiogenesis effect from the intersomitic blood vessels (ISVs) independent of high glucose [33]. Based on these previous reports, we incubated the zebrafish embryos in 500 *µ*M and 1000 *µ*M MG from 10 hpf (after gastrulation is completed) to 4 dpf to induce retinal vascular change and to mimic the diabetic conditions with elevated levels of MG with and without 30 mM glucose.

**Figure 2:**
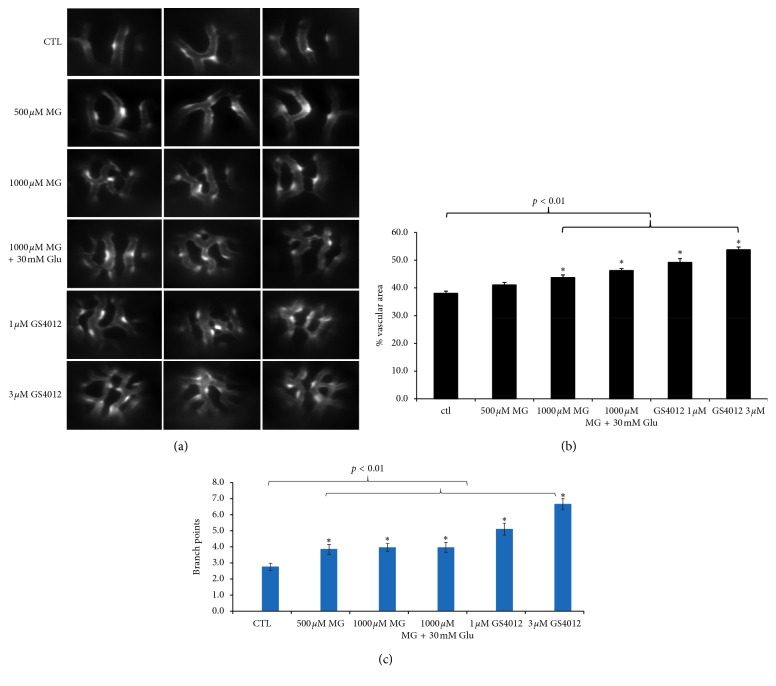
Effects of MG and glucose treatment on retinal angiogenesis in 4 dpf zebrafish. Zebrafish embryos were incubated with solution containing MG with or without glucose from 10 hpf to 4 dpf. (a) Fluorescence microscope observations of the retinal vessels of drug-treated embryos are shown at 4 dpf. The 1000 *µ*M MG treatment with or without 30 mM glucose group induced retinal vessel branching and vascular area compared with the untreated control in zebrafish embryos. As the positive control, zebrafish embryos treated with VEGF inducer GS4012 (1 *µ*M and 3 *µ*M) showed severe branching and disorganization in the retinal vasculature. Quantification of the percentage of vascular area (b) and the number of branch points (c) in midperiphral retina by MG, glucose, and GS4012 treatment in zebrafish embryos at 4 dpf. *n* = 30 eyes of 15 zebrafish embryos per group; mean ± SEM. ^*∗*^*p* < 0.05 and ^*∗∗*^*p* < 0.01 vs control group. Data were analyzed using the one-way ANOVA and then Student's *t*-test. Glu, glucose. Ctl, control.

Images obtained by an optical microscope did not show any gross morphological changes in MG-treated embryos and controls (data not shown). The fluorescent vasculature in the transgenic *TG(flk1:GFP)* zebrafish can be detected by fluorescence microscopy, and the vascular area of retina in the midperiphery was quantitated by Fiji-ImageJ [38] software in the 4 dpf embryos. When the zebrafish embryos were exposed to 1000 *µ*M MG with or without glucose, the retinal vessels in the midperiphery exhibited higher density in capillary networks resulted in increased vascular area than controls (Figure 2(a)). The percentage of vascular area and branch points was quantitated as shown in Figure 1 and found to be higher in the 1000 *µ*M MG group than that in the control group (*p* < 0.01) which indicated more angiogenesis after MG treatment, whereas in the 500 *µ*M MG group, the percentage of vascular area had no significant difference in comparison to the control (*p* > 0.05) (Figure 2(b)).

As to mimic the clinical situation of high glucose in patients with diabetes, in a group of 1000 *µ*M MG together with 30 mM glucose treatment, statistical analyses of the percentage of vascular area in retinal vessels also showed a significant increase in comparison to the control (*p* < 0.01) (Figure 2(b)). Moreover, it was also significantly higher in MG + glucose than that in the 1000 *µ*M MG group (*p* < 0.05). It is consistent with the result of the branch points of the retinal vessels that MG (500 *μ*M or 1000 *μ*M), MG + glu, and VEGF inducer (1 *μ*M or 3 *μ*M) were significantly higher than control (*p* < 0.01) (Figure 1(c)).

### 3.2. Effect of VEGF in Zebrafish Retinopathy

**Figure 3:**
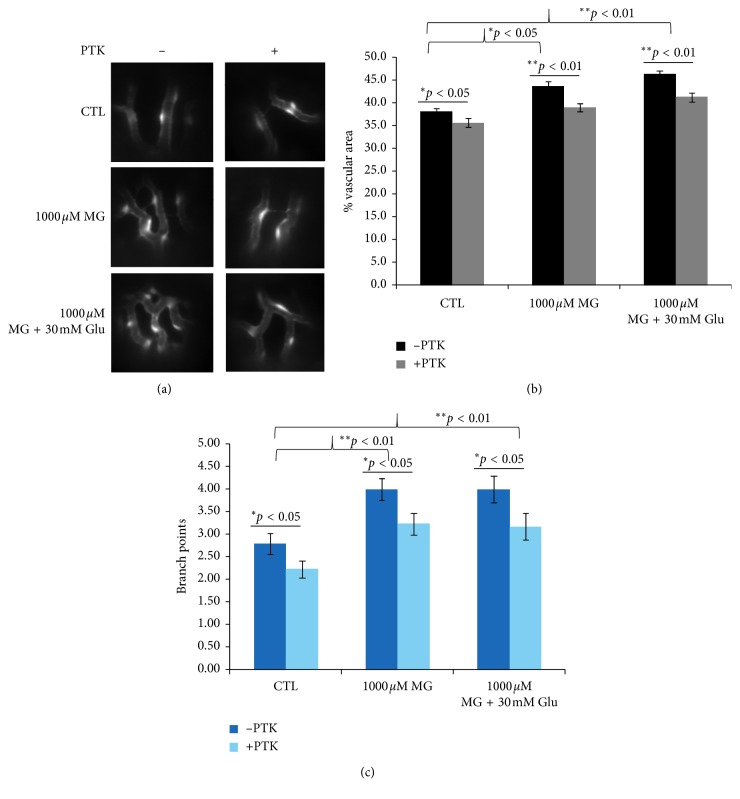
Effect of VEGF receptor inhibitor (PTK787) in MG-induced zebrafish. (a) Fluorescence microscope observations shown added 0.5 *µ*M PTK787 in 1 dpf in the 1000 *µ*M MG and 30 mM glucose treatment group reverted blood vessel hyperbranching in zebrafish embryos at 4 dpf. The proangiogenic effect induced by MG treatment was inhibited and exhibited no significant difference compared with the no-treatment control. Quantification of the percentage of vascular area (b) and number of branch points (c) in midperiphral retina by PTK787, MG, and glucose treatment in zebrafish embryos at 4 dpf. *n* = 30 eyes of 15 zebrafish embryos per group; mean ± SEM. ^*∗*^*p*  <  0.05, ^*∗∗*^*p* < 0.01 vs control group. *p* > 0.05 nonsignificant (NS). Data were analyzed using the one-way ANOVA and then Student's *t*-test. Glu, glucose. CTL, control.

As a positive control, GS4012, a VEGF inducer showed a prominent sign of increased vessel branching, similar to MG and glucose treatment, indicating dose-dependent angiogenic response as expected in both the treatment groups at 1 *µ*M and 3 *µ*M (Figures 2(a) and 2(b)). VEGF plays an important role in the pathogenesis of DR through angiogenesis by binding to its receptor VEGFR2 in endothelial cells [6, 39]. In order to assess the role of VEGFR2 in our MG-induced neovascular retinopathy model, a VEGFR tyrosine kinase inhibitor PTK787 (0.5 *µ*M) was used to evaluate its effect on MG-induced angiogenesis, from 1 to 4 dpf. As shown in Figure 3(a), the vascular branches reduced significantly in MG + 0.5 *µ*M PTK787 when compared to that in the MG group with or without glucose. The percentage of vascular area in the “1000 *µ*M MG + 0.5 *µ*M PTK787” group showed a rescue in retinopathy or a significant decrease of retinal angiogenesis than 1000 *µ*M MG group (*p* < 0.01) and had no statistical difference than the control. Similarly, when 0.5 *µ*M PTK787 was added to 1000 *µ*M MG or 1000 *µ*M MG + 30 mM glucose treatment groups, the percentage of vascular area decreased significantly (*p* < 0.01) and returned back to a level similar to CTL (Figure 3(b)). Similarly, the branch points were also significantly reduced after PTK787 treatment to CTL, MG, and MG + Glu groups (Figure 3(c)).

**Figure 4:**
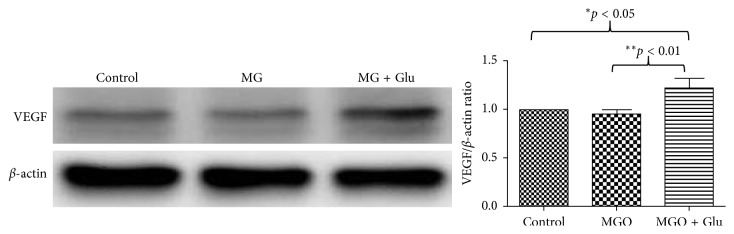
Western blot analysis of VEGF expression in zebrafish eyes after MG and glucose treatments. Equal amounts of zebrafish eye extracts were analyzed by western blot using anti-VEGF and anti-actin antibodies, from three independent experiments. The immunoblot bands were quantified by densitometry analysis using ImageJ software, and the ratio to *β*-actin was calculated by setting the value of control as 1. The resulting relative values of VEGF are shown in histograms (right panel). Values are presented as mean ± SEM. Significant difference analyzed using the one-way ANOVA and then Student's *t*-test: ^*∗*^*p* < 0.05; ^*∗∗*^*p* < 0.01.

These findings indicated the addition of the VEGFR inhibitor abolished the proangiogenic effects of 1000 *µ*M MG treatment with or without 30 mM glucose. We argued that the effect of MG and glucose may be mediated by induction of VEGF. Therefore, we dissected the whole eyes from 4 dpf embryos from control and 1000 *µ*M MG with or without 30 mM glucose and homogenized in RIPA extraction buffer for western blot analysis. We detected a significant increase in the amount of VEGF protein in the eyes after MG + glucose treatment (Figure 4, western blot). However, the amount of VEGF protein in the eyes of the MG alone group was not significantly higher than that in the control. Although the western blot cannot detect a higher level of VEGF protein, we cannot exclude the VEGF-VEGFR signaling axis involved because the VEGFR kinase inhibitor PTK787 can inhibit the proangiogenic effect of MG and MG + glu in the retina. We speculate the positive feedback mechanism of VEGF-VEGFR axis is regulated by MG or MG + glu but the protein level of VEGF is least affected by MG alone.

## 4. Discussion

Several studies reported MG elevation in blood and tissues of diabetic patients and animals [26, 39, 40]. MG has been considered a causative factor for retinal vascular injury [26, 39]. The possible mechanism has been implied by the early studies: MG leads to the damage of the blood-retinal barrier by disrupting the tight junction protein [41] which is associated with diabetic microvascular pathology [20, 42]. MG also induced apoptosis of bovine and rat retinal pericytes [39, 43], in association with the formation of acellular capillaries [44, 45]. These changes lead to the formation of nonperfusion and ischemia of retina and increase in the possibility of retinal angiogenesis. Jorgens et al. showed that external MG can increase the bioavailability of MG in zebrafish embryos and MG treatment (200 or 500 *µ*M) can induce angiogenesis of the trunk intersomitic blood vessels in zebrafish embryos within only 4 days [33]. Culturing in 30 mM glucose solution led to a threefold increase of MG in zebrafish embryos, and high tissue glucose increased tissue MG concentrations in zebrafish at 96 hpf. In addition, they found that incubation with MG or glucose did not affect structure of major blood vessels, including the dorsal aorta (DA) in the zebrafish embryos. The altered vascular hyperbranches were observed only in ISV of trunk regions starting at 72 hpf. By contrast, our study focused on the retinal vasculature in the midperiphery and not the central bigger vessels of the retina. Our goal is to study short exposure time with 500 *µ*M and 1000 *µ*M MG and its potential role in the pathogenesis of diabetic retinopathy and induced retinal angiogenesis as a potential neovascular retinopathy zebrafish model.

Our study found an increase of the retinal vascular area in 4 dpf embryos when treated with 1000 *µ*M MG in comparison to the control indicated abnormal retinal angiogenesis. These results are comparable to the similar findings that MG might display a dosage-dependent manner after exogenous methylglyoxal treatment [33]. To mimic the clinical condition of diabetic patients, 1000 *µ*M MG + 30 mM glucose was shown effective leading to increased retinal vascular area. The angiogenesis effect with the addition of 30 mM glucose was indeed induced even stronger than 1000 *µ*M MG treatment alone. These findings raised the possibility that elevated blood glucose and increased MG in vivo may increase the risk for development of diabetic complication of retinal vascular angiogenesis.

Many species of animal models have been established to examine the pathogenic mechanism or to evaluate therapeutics against DR. However, slow response limits the usefulness for drug screening, such as 17 weeks for retinal angiogenic changes in STZ-induced diabetic mice after the onset of hyperglycemia [46] and 14–21 days for neovascularization in rat eyes after intravitreal injection of VEGF [47]. By contrast, hypoxia-induced retinal angiogenic responses in zebrafish can be detected after only 3 days [48]. Here, we showed proangiogenic effect of MG was observed in 4 dpf and we suggested that zebrafish might be a potential alternative to study the effect of MG on DR. We argue that zebrafish embryos may provide a distinctive and competitive advantage complimented to other animal models, such as large-scale small-molecule screen, automation for high-resolution in vivo screen, studying the early event, shorter experimental time, and real-time live observation [49–51]. It was shown that high glucose treatment in zebrafish induced expression of VEGF [52]. VEGF is considered to be the primary factor of pathological angiogenesis in the retina of DR [53]. Our study showed that a known VEGFR inhibitor, PTK787, reduced the severity of MG-induced retinopathy. Our results are consistent with Jorgens and colleagues that MG is proangiogenic in retina via VEGF-VEGFR signaling pathway in the zebrafish. Based on the angiogenesis effect of MG, future study of using MG-induced zebrafish model may provide a feasible animal model to screen for bioactive compounds to alleviate the risk of neovascular retinopathy.

## Data Availability

The data used to support the findings of this study are included within the article.

## Abbreviations

MG: Methylglyoxal
AGEs: Advanced glycation end products
PDR: Proliferative diabetic retinopathy
NPDR: Nonproliferative diabetic retinopathy
hpf: Hour-post-fertilization
dpf: Day-post-fertilization
DM: Diabetes mellitus
VEGF: Vascular endothelial growth factor
flk1 or VEGFR2: Vascular endothelial growth factor receptor 2
GFP: Green fluorescent protein
*Tg(flk1:GFP)*: Vascular specific transgenic zebrafish line expressing fluorescent GFP in vasculature.

## References

[1] Klein R. (1988). Glycosylated hemoglobin predicts the incidence and progression of diabetic retinopathy. *JAMA*.

[2] Wong T. Y., Klein R., Islam F. M. A. (2006). Diabetic retinopathy in a multi-ethnic cohort in the United States. *American Journal of Ophthalmology*.

[3] Rathmann W., Giani G. (2004). Global prevalence of diabetes: estimates for the year 2000 and projections for 2030: response to wild et al.. *Diabetes Care*.

[4] Morello C. M. (2007). Etiology and natural history of diabetic retinopathy: an overview. *American Journal of Health-System Pharmacy*.

[5] Malone J. I., Morrison A. D., Pavan P. R., Cuthbertson D. D. (2001). Prevalence and significance of retinopathy in subjects with type 1 diabetes of less than 5 years’ duration screened for the diabetes control and complications trial. *Diabetes Care*.

[6] Caldwell R. B., Bartoli M., Behzadian M. A. (2003). Vascular endothelial growth factor and diabetic retinopathy: pathophysiological mechanisms and treatment perspectives. *Diabetes/Metabolism Research and Reviews*.

[7] Jung S.-H., Kim Y. S., Lee Y.-R., Kim J. S. (2016). High glucose-induced changes in hyaloid-retinal vessels during early ocular development of zebrafish: a short-term animal model of diabetic retinopathy. *British Journal of Pharmacology*.

[8] Neuhauss S. C. F. (2003). Behavioral genetic approaches to visual system development and function in zebrafish. *Journal of Neurobiology*.

[9] Brockerhoff S. E., Hurley J. B., Janssen-Bienhold U., Neuhauss S. C., Driever W., Dowling J. E. (1995). A behavioral screen for isolating zebrafish mutants with visual system defects. *Proceedings of the National Academy of Sciences*.

[10] Neuhauss S. C. F., Biehlmaier O., Seeliger M. W. (1999). Genetic disorders of vision revealed by a behavioral screen of 400 essential loci in zebrafish. *Journal of Neuroscience*.

[11] Bilotta J., Saszik S. (2001). The zebrafish as a model visual system. *International Journal of Developmental Neuroscience*.

[12] Gerhard G. S., Kauffman E. J., Wang X. (2002). Life spans and senescent phenotypes in two strains of zebrafish (Danio rerio). *Experimental Gerontology*.

[13] Fleisch V. C., Neuhauss S. C. F. (2006). Visual behavior in zebrafish. *Zebrafish*.

[14] Roman B. L., Weinstein B. M. (2000). Building the vertebrate vasculature: research is going swimmingly. *Bioessays*.

[15] Shin J. T., Fishman M. C. (2002). FROMZEBRAFISH TOHUMAN: modular medical models. *Annual Review of Genomics and Human Genetics*.

[16] Rubinstein A. L. (2003). Zebrafish: from disease modeling to drug discovery. *Current Opinion in Drug Discovery and Development*.

[17] Alvarez Y., Cederlund M. L., Cottell D. C. (2007). Genetic determinants of hyaloid and retinal vasculature in zebrafish. *BMC Developmental Biology*.

[18] Rouhi P., Lee S. L. C., Cao Z., Hedlund E.-M., Jensen L. D., Cao Y. (2010). Pathological angiogenesis facilitates tumor cell dissemination and metastasis. *Cell Cycle*.

[19] Lee S. L. C., Rouhi P., Jensen L. D. (2009). Hypoxia-induced pathological angiogenesis mediates tumor cell dissemination, invasion, and metastasis in a zebrafish tumor model. *Proceedings of the National Academy of Sciences*.

[20] Lapolla A., Flamini R., Vedova A. D. (2003). Glyoxal and methylglyoxal levels in diabetic patients: quantitative determination by a new GC/MS method. *Clinical Chemistry and Laboratory Medicine*.

[21] Kilhovd B. K., Giardino I., Torjesen P. A. (2003). Increased serum levels of the specific AGE-compound methylglyoxal-derived hydroimidazolone in patients with type 2 diabetes. *Metabolism*.

[22] Stitt A., Frizzell N., Thorpe S. (2004). Advanced glycation and advanced lipoxidation: possible role in initiation and progression of diabetic retinopathy. *Current Pharmaceutical Design*.

[23] Morgan P. E., Sheahan P. J., Davies M. J. (2014). Perturbation of human coronary artery endothelial cell redox state and NADPH generation by methylglyoxal. *PLoS One*.

[24] Choi Y. Y., Kim S., Han J.-H. (2016). CHOP deficiency inhibits methylglyoxal-induced endothelial dysfunction. *Biochemical and Biophysical Research Communications*.

[25] Wu L., Juurlink B. H. J. (2002). Increased methylglyoxal and oxidative stress in hypertensive rat vascular smooth muscle cells. *Hypertension*.

[26] Miller A. G., Smith D. G., Bhat M., Nagaraj R. H. (2006). Glyoxalase I is critical for human retinal capillary pericyte survival under hyperglycemic conditions. *Journal of Biological Chemistry*.

[27] Ahmed N., Thornalley P. J., Dawczynski J. (2003). Methylglyoxal-derived hydroimidazolone advanced glycation end-products of human lens proteins. *Investigative Opthalmology & Visual Science*.

[28] Karachalias N., Babaei-Jadidi R., Ahmed N., Thornalley P. J. (2003). Accumulation of fructosyl-lysine and advanced glycation end products in the kidney, retina and peripheral nerve of streptozotocin-induced diabetic rats. *Biochemical Society Transactions*.

[29] Fosmark D. S., Bragadóttir R., Stene-Johansen I. (2007). Increased vitreous levels of hydroimidazolone in type 2 diabetes patients are associated with retinopathy: a case-control study. *Acta Ophthalmologica Scandinavica*.

[30] Fosmark D. S., Torjesen P. A., Kilhovd B. K. (2006). Increased serum levels of the specific advanced glycation end product methylglyoxal-derived hydroimidazolone are associated with retinopathy in patients with type 2 diabetes mellitus. *Metabolism*.

[31] Gestri G., Link B. A., Neuhauss S. C. (2012). The visual system of zebrafish and its use to model human ocular diseases. *Developmental Neurobiology*.

[32] Chhetri J., Jacobson G., Gueven N. (2014). Zebrafish–on the move towards ophthalmological research. *Eye*.

[33] Jörgens K., Stoll S. J., Pohl J. (2015). High tissue glucose alters intersomitic blood vessels in zebrafish via methylglyoxal targeting the VEGF receptor signaling cascade. *Diabetes*.

[34] Weinstein B. M., Schier A. F., Abdelilah S. (1996). Hematopoietic mutations in the zebrafish. *Development*.

[35] Peterson R. T., Shaw S. Y., Peterson T. A. (2004). Chemical suppression of a genetic mutation in a zebrafish model of aortic coarctation. *Nature Biotechnology*.

[36] Hamanaka T., Akabane N., Yajima T., Takahashi T., Tanabe A. (2001). Retinal ischemia and angle neovascularization in proliferative diabetic retinopathy. *American Journal of Ophthalmology*.

[37] Kim J., Son J.-W., Lee J.-A., Oh Y.-S., Shinn S.-H. (2004). Methylglyoxal induces apoptosis mediated by reactive oxygen species in bovine retinal pericytes. *Journal of Korean Medical Science*.

[38] Schindelin J., Arganda-Carreras I., Frise E. (2012). Fiji: an open-source platform for biological-image analysis. *Nature Methods*.

[39] Scheppke L., Aguilar E., Gariano R. F. (2008). Retinal vascular permeability suppression by topical application of a novel VEGFR2/Src kinase inhibitor in mice and rabbits. *Journal of Clinical Investigation*.

[40] Randell E. W., Vasdev S., Gill V. (2005). Measurement of methylglyoxal in rat tissues by electrospray ionization mass spectrometry and liquid chromatography. *Journal of Pharmacological and Toxicological Methods*.

[41] Kim J., Kim C.-S., Lee Y. M., Jo K., Shin S. D., Kim J. S. (2012). Methylglyoxal induces hyperpermeability of the blood-retinal barrier via the loss of tight junction proteins and the activation of matrix metalloproteinases. *Graefe’s Archive for Clinical and Experimental Ophthalmology*.

[42] Beisswenger P. J., Howell S. K., Touchette A. D., Lal S., Szwergold B. S. (1999). Metformin reduces systemic methylglyoxal levels in type 2 diabetes. *Diabetes*.

[43] Kim O. S., Kim J., Kim C.-S., Kim N. H., Kim J. S. (2010). KIOM-79 prevents methyglyoxal-induced retinal pericyte apoptosis in vitro and in vivo. *Journal of Ethnopharmacology*.

[44] Hammes H.-P. (2003). Pathophysiological mechanisms of diabetic angiopathy. *Journal of Diabetes and its Complications*.

[45] Hammes H.-P., Du X., Edelstein D. (2003). Benfotiamine blocks three major pathways of hyperglycemic damage and prevents experimental diabetic retinopathy. *Nature Medicine*.

[46] Su L., Ji J., Bian J., Fu Y., Ge Y., Yuan Z. (2012). Tacrolimus (FK506) prevents early retinal neovascularization in streptozotocin-induced diabetic mice. *International Immunopharmacology*.

[47] Helfenstein T., Fonseca F. A., Ihara S. S. (2011). Impaired glucose tolerance plus hyperlipidaemia induced by diet promotes retina microaneurysms in New Zealand rabbits. *International Journal of Experimental Pathology*.

[48] Cao R., Jensen L. D. E., Söll I., Hauptmann G., Cao Y. (2008). Hypoxia-induced retinal angiogenesis in zebrafish as a model to study retinopathy. *PLoS One*.

[49] Hao J., Williams C. H, Webb M. E, Hong C. C (2010). Large scale zebrafish-based in vivo small molecule screen. *Journal of Visualized Experiments*.

[50] Trompouki E., Zon L. I. (2010). Small molecule screen in zebrafish and HSC expansion. *Cellular Programming and Reprogramming*.

[51] Early J. J., Cole K. L. H., Williamson J. M. (2018). An automated high-resolution in vivo screen in zebrafish to identify chemical regulators of myelination. *Elife*.

[52] Alvarez Y., Chen K., Reynolds A. L., Waghorne N., O’Connor J. J., Kennedy B. N. (2010). Predominant cone photoreceptor dysfunction in a hyperglycaemic model of non-proliferative diabetic retinopathy. *Disease Models & Mechanisms*.

[53] Ozaki H., Seo M.-S., Ozaki K. (2000). Blockade of vascular endothelial cell growth factor receptor signaling is sufficient to completely prevent retinal neovascularization. *American Journal of Pathology*.

